# Supplementary material for: Procaine Abrogates the Epithelial-Mesenchymal Transition Process through Modulating c-Met Phosphorylation in Hepatocellular Carcinoma
Source: Cancers (Basel). 2022 Oct 11;14(20):4978. doi: 10.3390/cancers14204978 (PMC9599628; doi:10.3390/cancers14204978)
Supplement: Supplementary file 1 [file cancers-14-04978-s001.zip › cancers-1894158-supplementary.pdf]

A.

S1

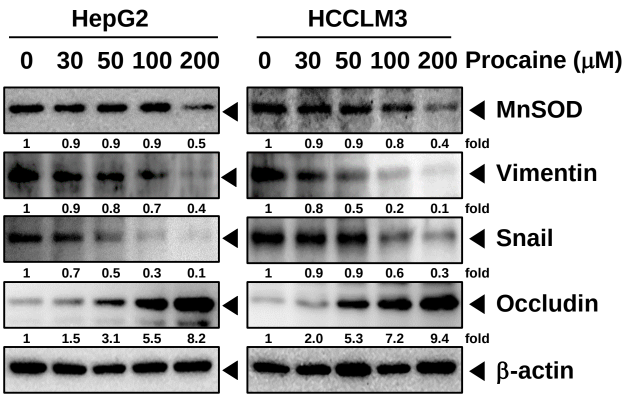
HepG2
HCCLM3
0 30 50 100 200 0 30 50 100 200 Procaine (μM)
MnSOD
1 0.9 0.9 0.9 0.5 1 0.9 0.9 0.8 0.4 fold
Vimentin
1 0.9 0.8 0.7 0.4 1 0.8 0.5 0.2 0.1 fold
Snail
1 0.7 0.5 0.3 0.1 1 0.9 0.9 0.6 0.3 fold
Occludin
1 1.5 3.1 5.5 8.2 1 2.0 5.3 7.2 9.4 fold
β-actin

Fig.S1 A

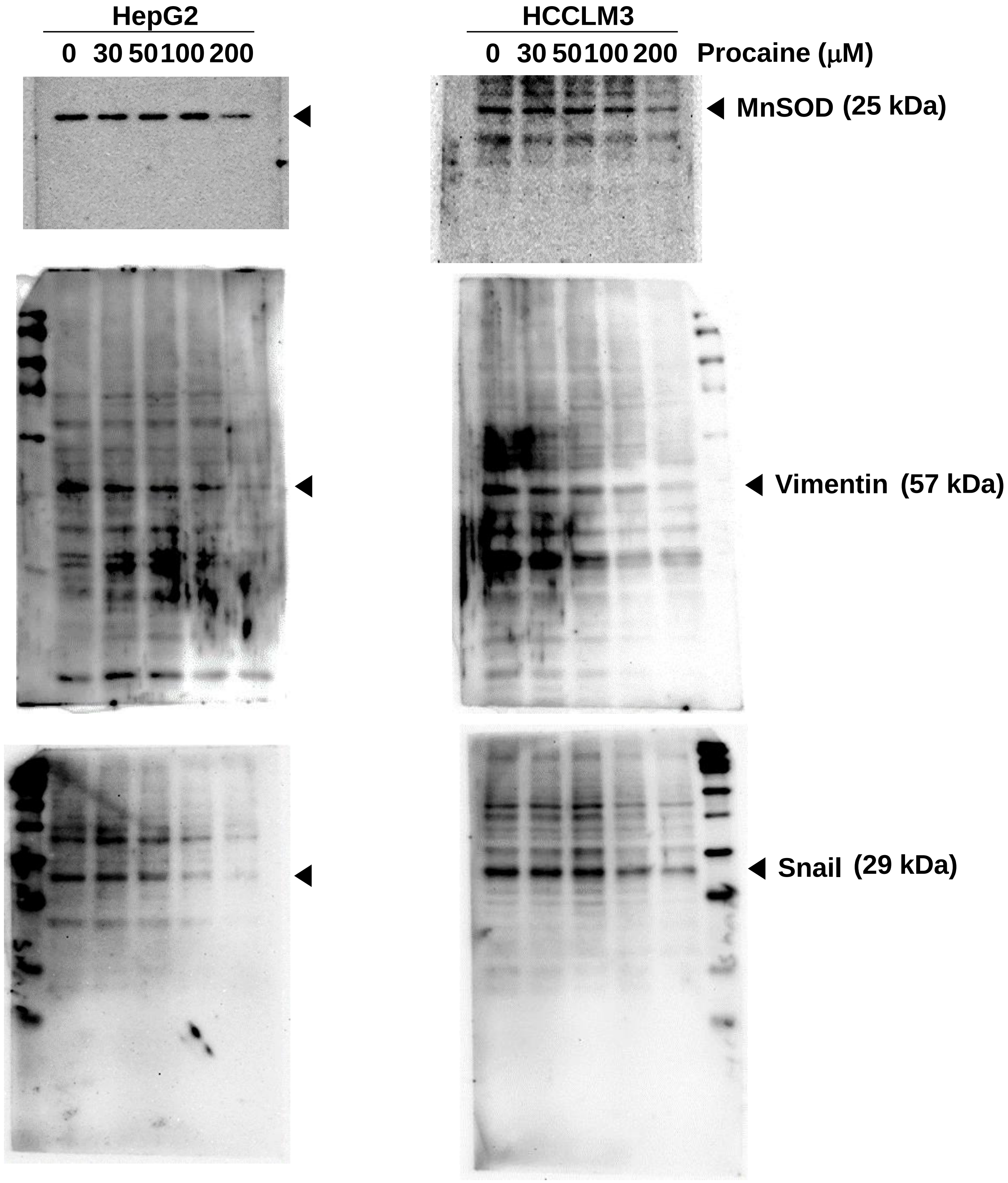

HepG2
HCCLM3
0 30 50 100 200
0 30 50 100 200
Procaine (μM)
MnSOD (25 kDa)
Vimentin (57 kDa)
Snail (29 kDa)

Fig.S1 A

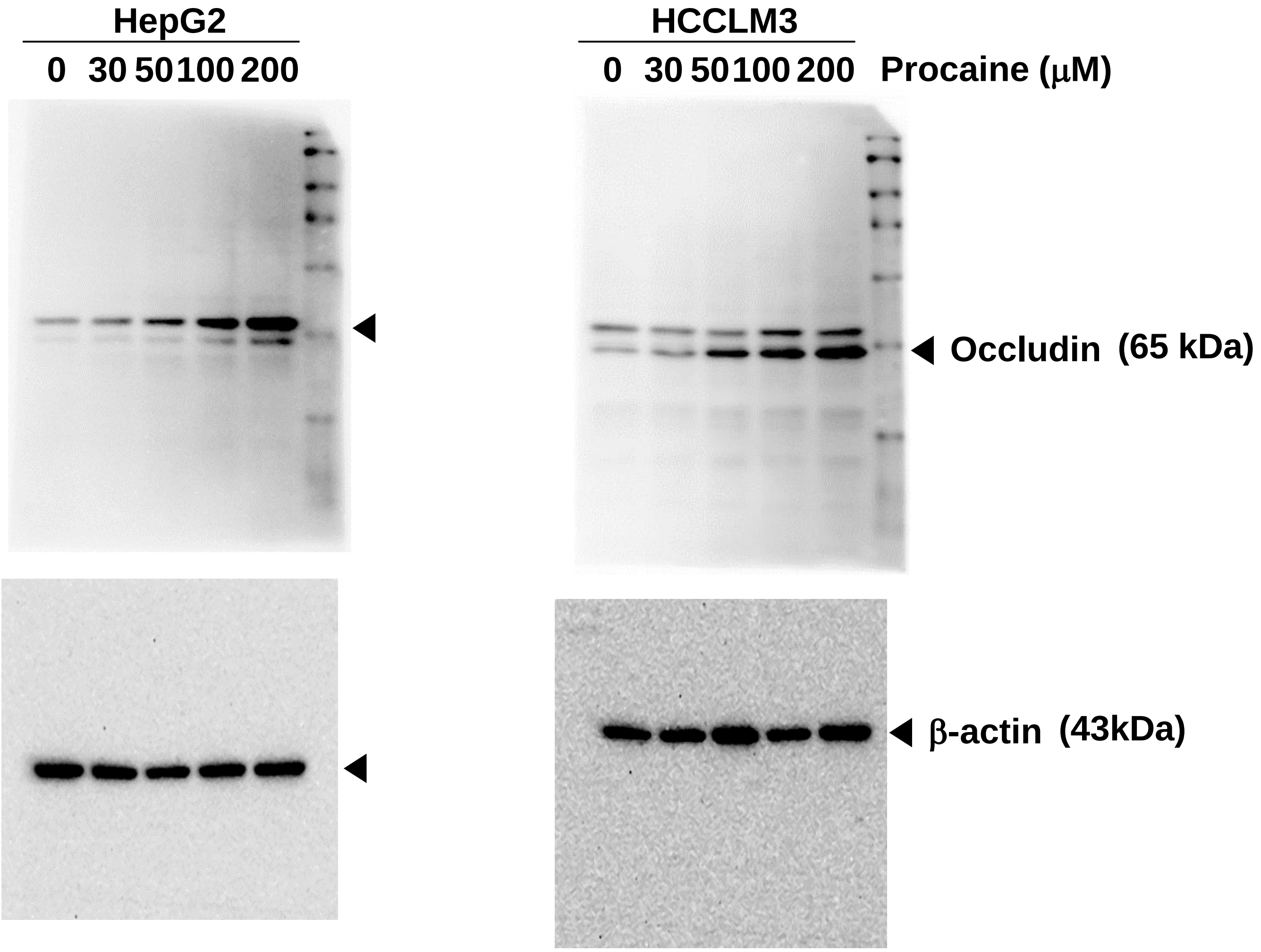
HepG2
0
30
50
100
200
HCCLM3
0
30
50
100
200
Procaine (μM)
Occludin (65 kDa)
β-actin (43kDa)

Fig.1C

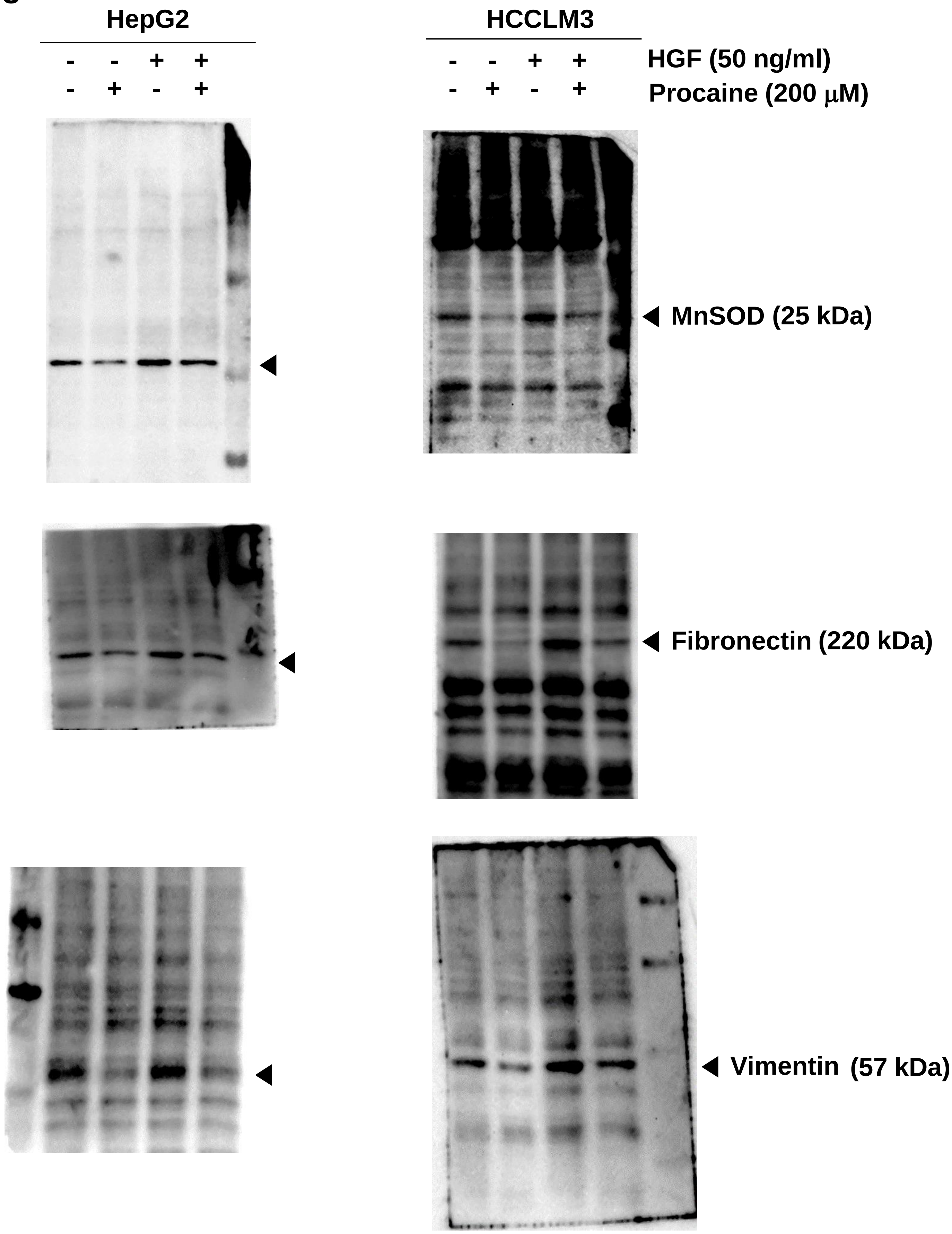
HepG2
HCCLM3
- - + + HGF (50 ng/ml)
- + - + Procaine (200 μM)
MnSOD (25 kDa)
Fibronectin (220 kDa)
Vimentin (57 kDa)

Fig.1C

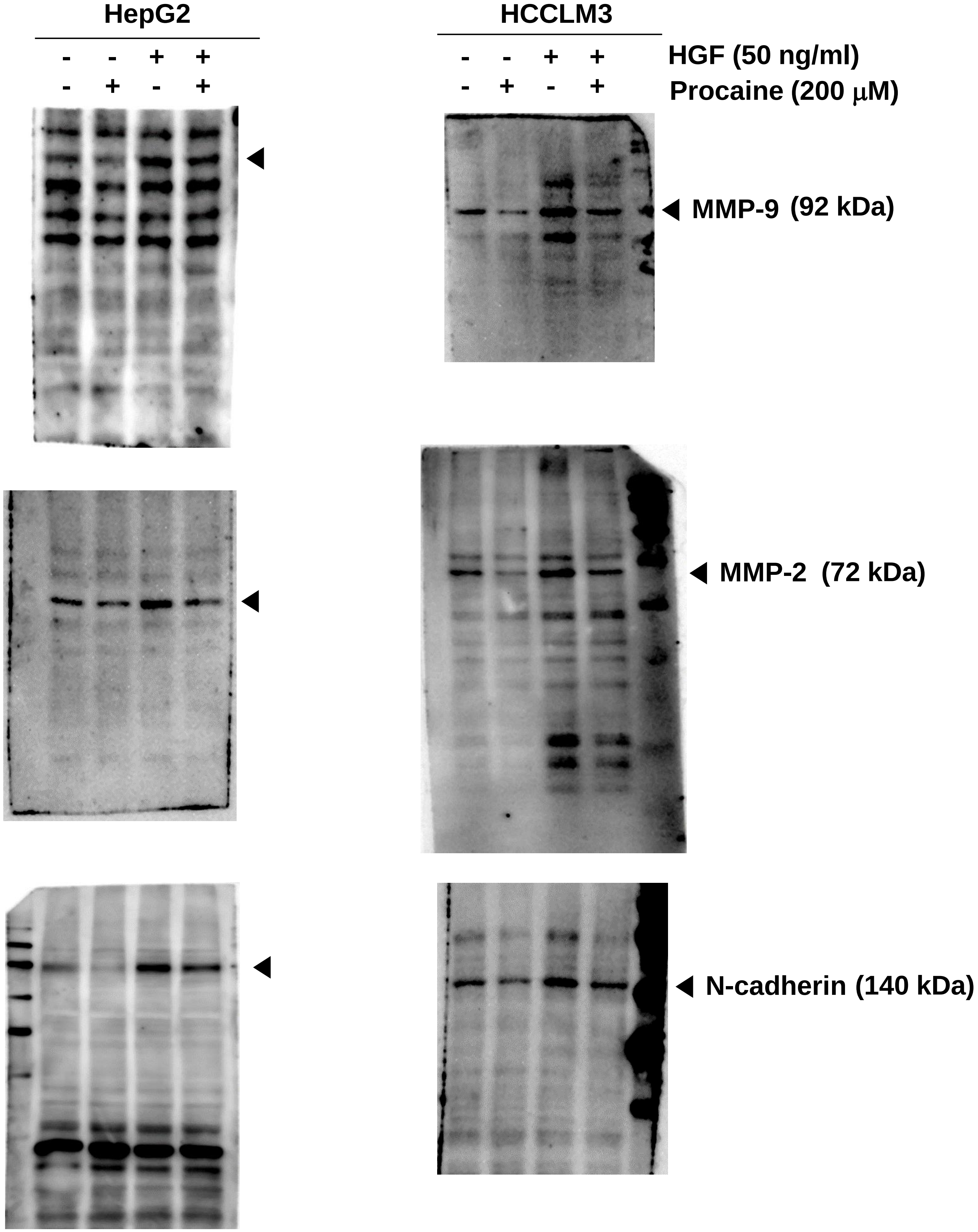
HepG2
HCCLM3
HGF (50 ng/ml)
Procaine (200 μM)
MMP-9 (92 kDa)
MMP-2 (72 kDa)
N-cadherin (140 kDa)

Fig.1C

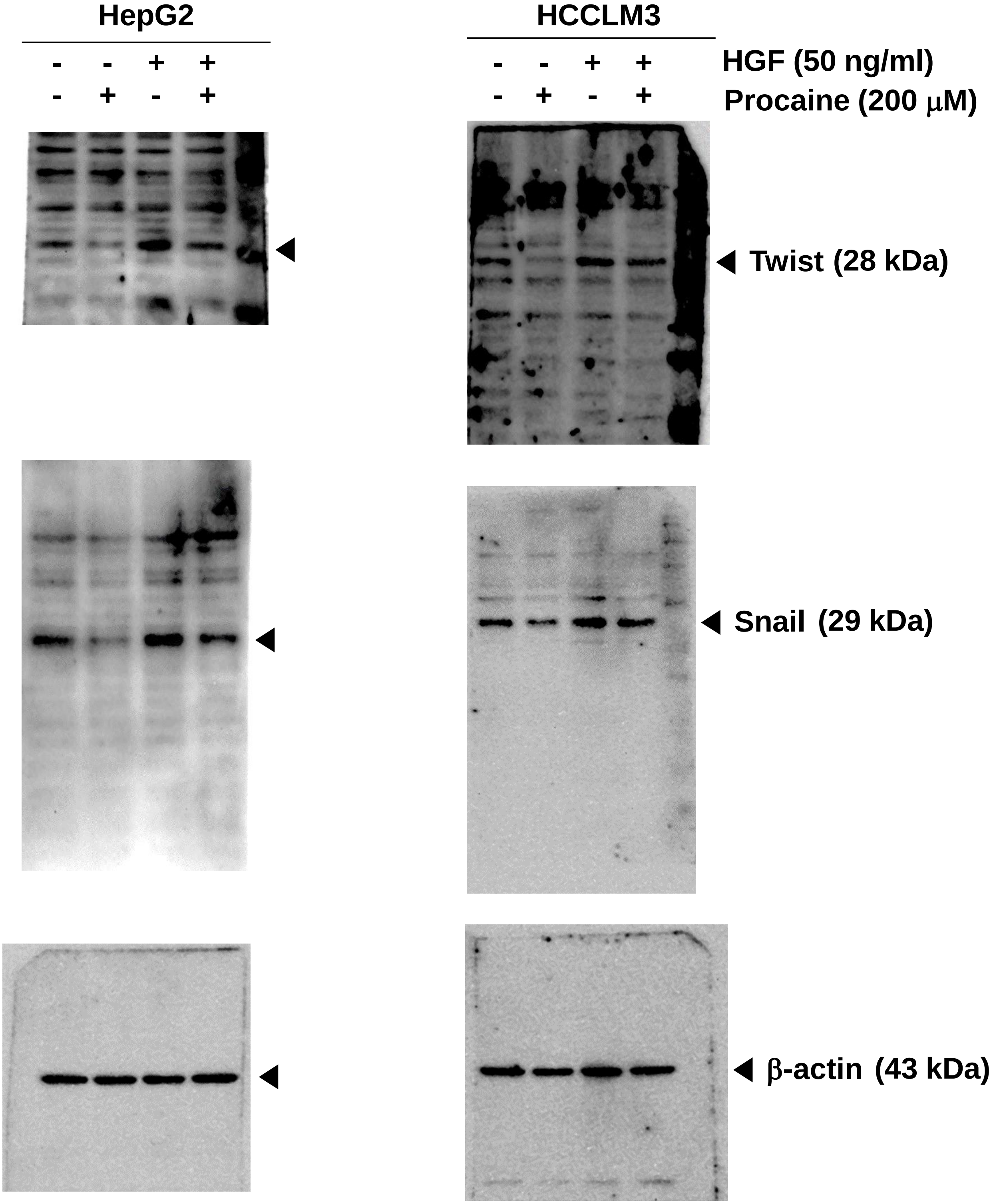
HepG2
HCCLM3
HGF (50 ng/ml)
Procaine (200 μM)
Twist (28 kDa)
Snail (29 kDa)
β-actin (43 kDa)

Fig.1D

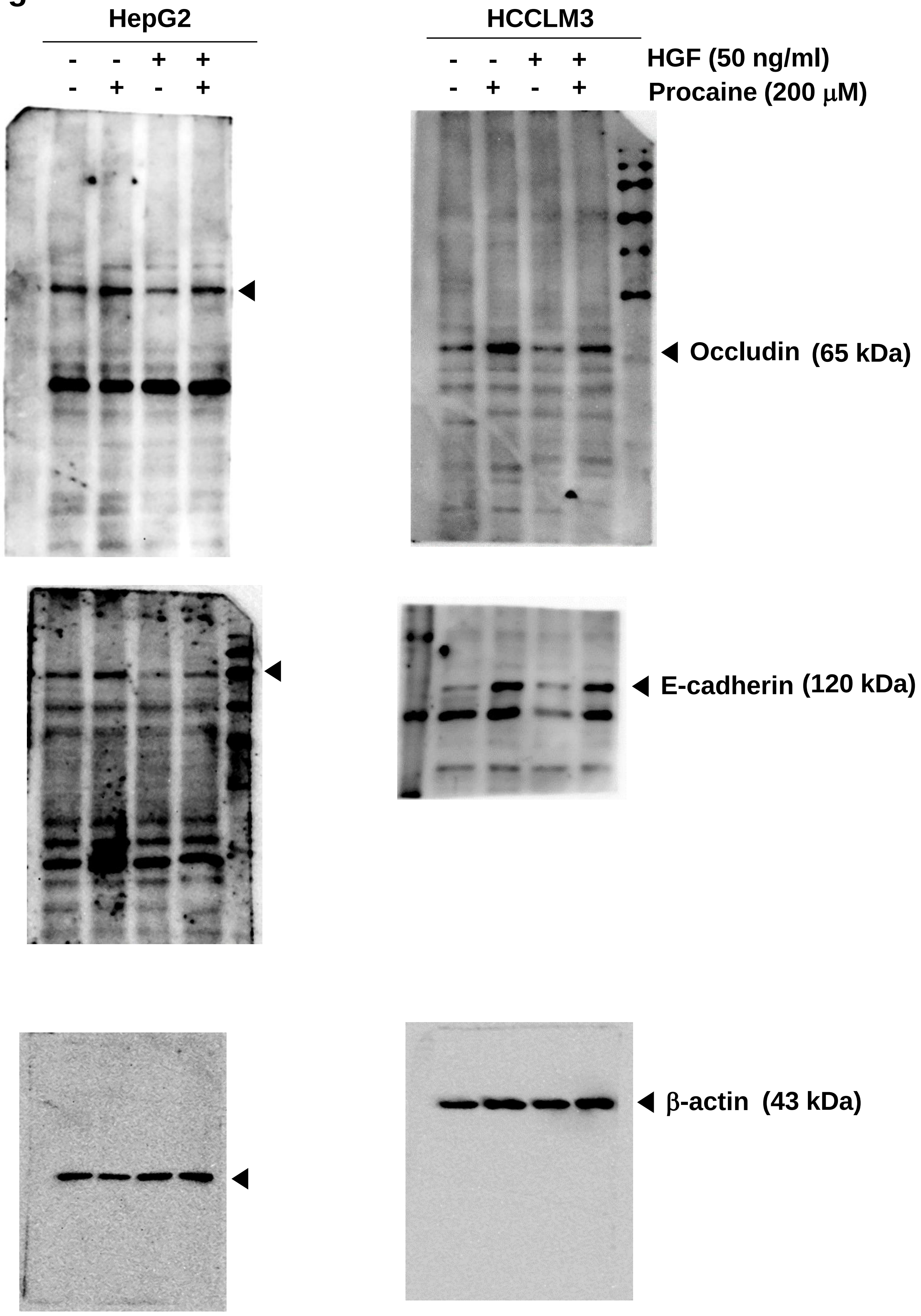
HepG2
HCCLM3
- - + +
- + - +
- - + +
- + - +
HGF (50 ng/ml)
Procaine (200 μM)
Occludin (65 kDa)
E-cadherin (120 kDa)
β-actin (43 kDa)

Fig.3A

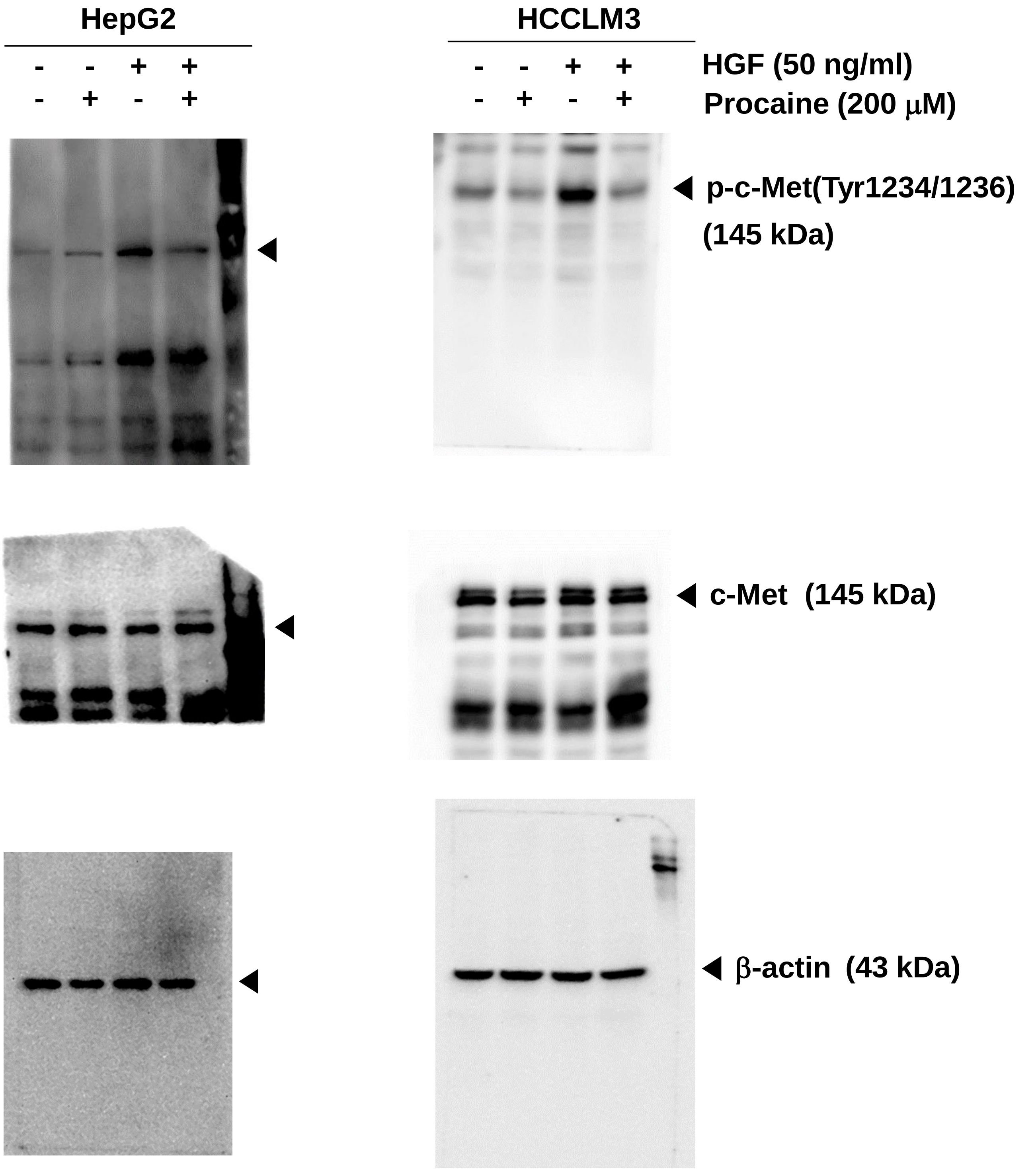
HepG2
HCCLM3
- - + +
- + - +
- - + +
- + - +
HGF (50 ng/ml)
Procaine (200 μM)
p-c-Met(Tyr1234/1236)
(145 kDa)
c-Met (145 kDa)
β-actin (43 kDa)

Fig.3B

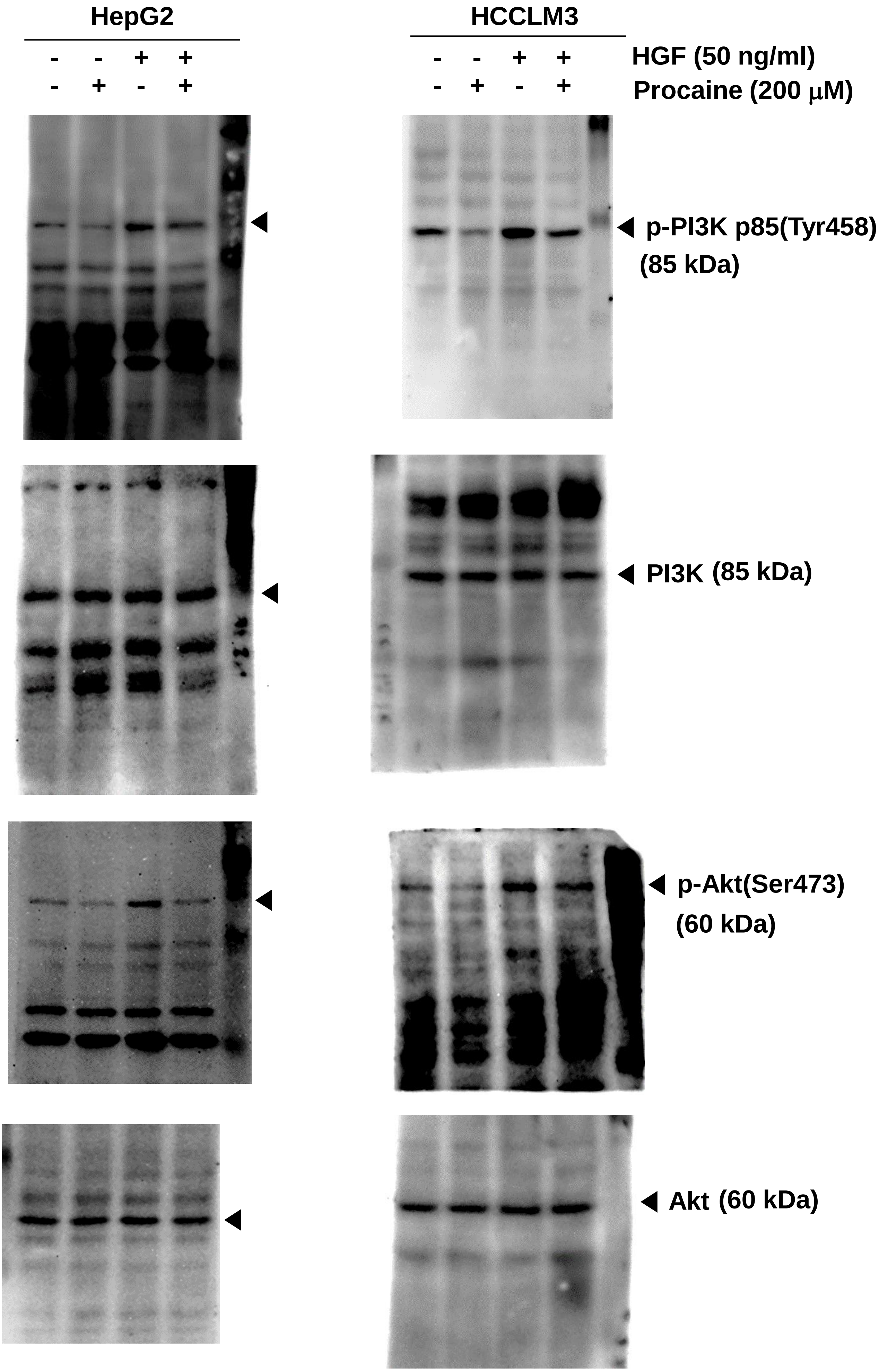
HepG2
HCCLM3
- - + +
- + - +
HGF (50 ng/ml)
Procaine (200 μM)
p-PI3K p85(Tyr458)
(85 kDa)
PI3K (85 kDa)
p-Akt(Ser473)
(60 kDa)
Akt (60 kDa)

Fig.3B

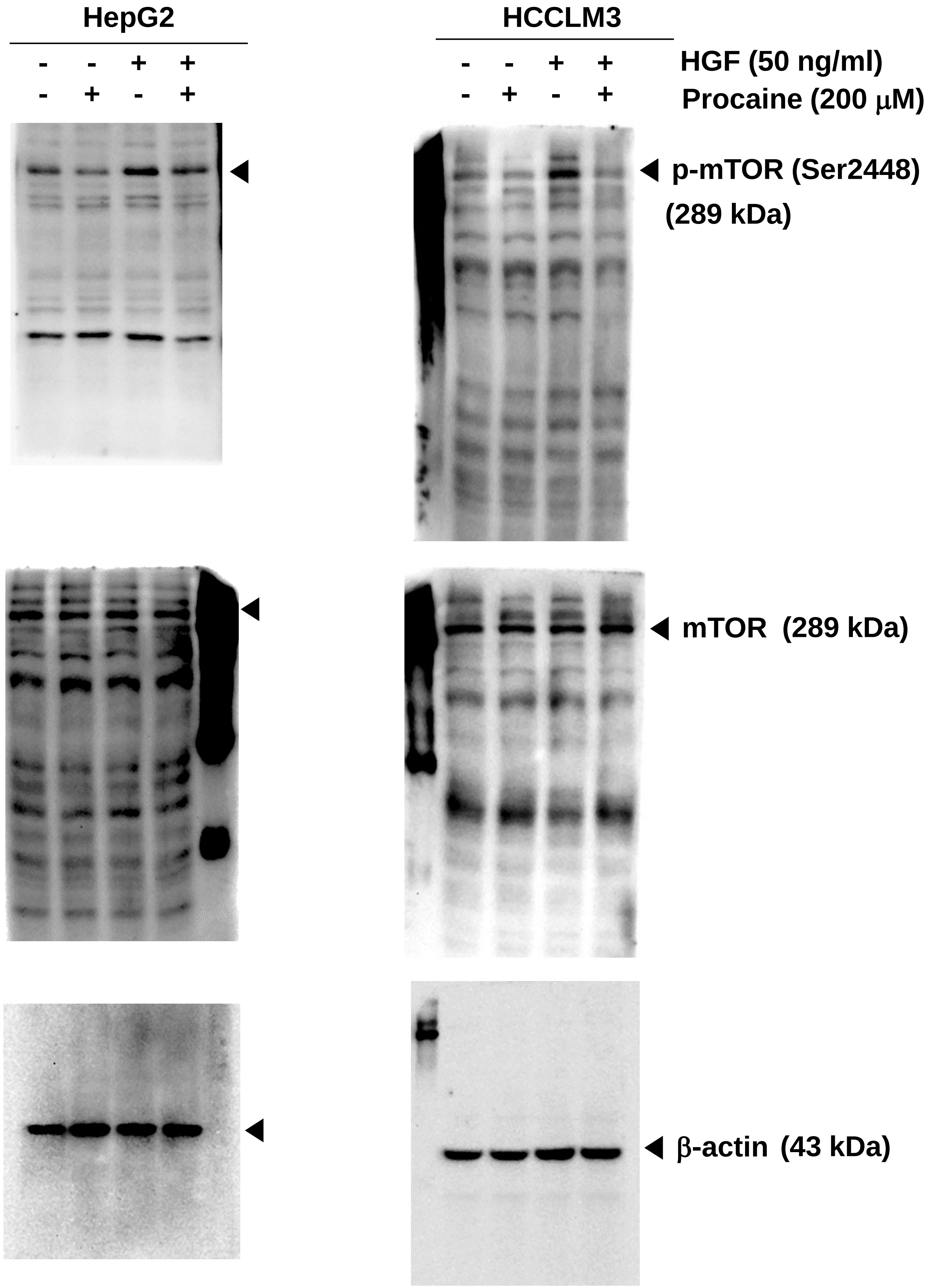
HepG2
HCCLM3
- - + +
- + - +
- - + +
- + - +
HGF (50 ng/ml)
Procaine (200 μM)
p-mTOR (Ser2448)
(289 kDa)
mTOR (289 kDa)
β-actin (43 kDa)

Fig.3C

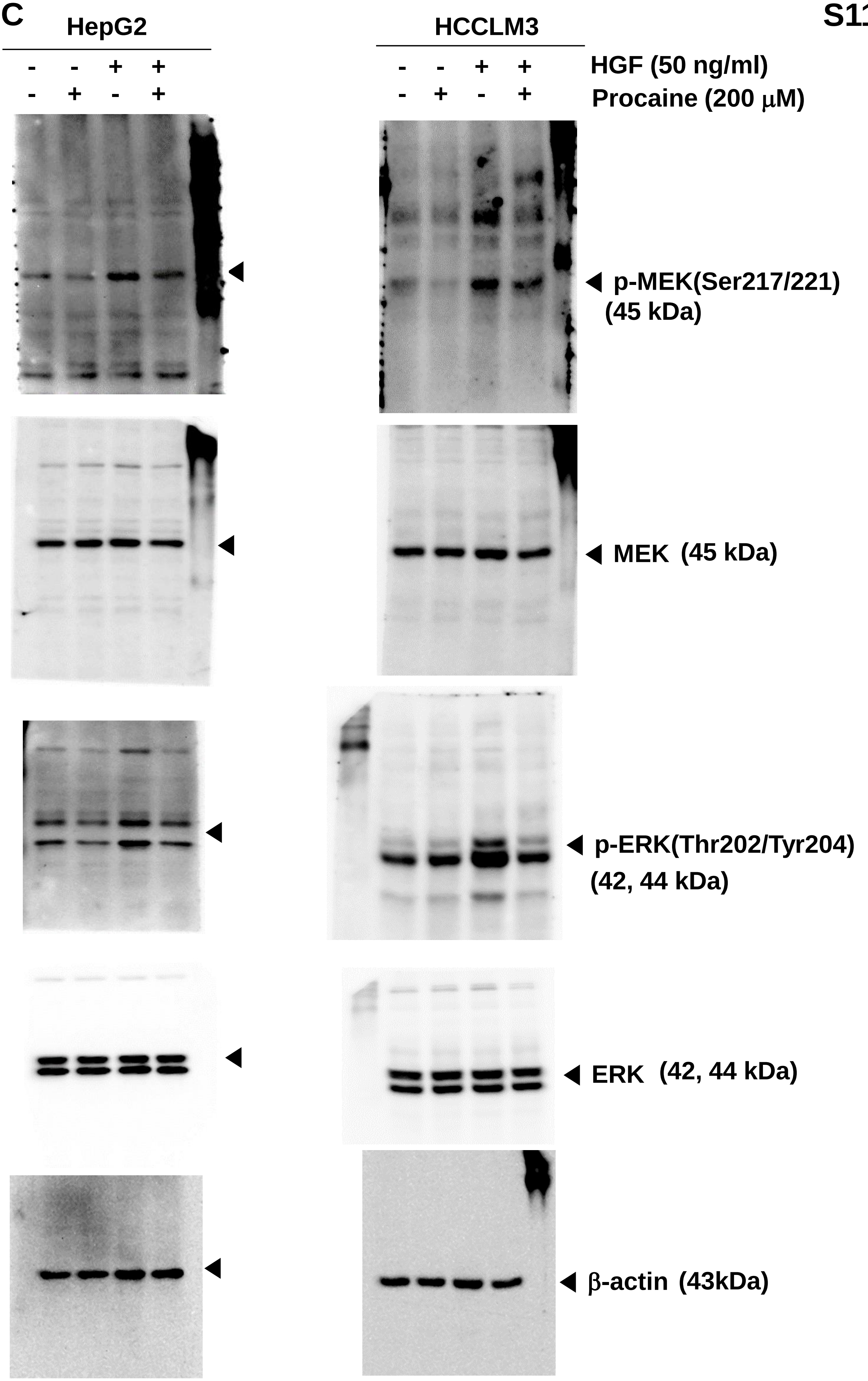

HepG2
HCCLM3
- - + +
- + - +
HGF (50 ng/ml)
Procaine (200 μM)
p-MEK(Ser217/221)
(45 kDa)
MEK (45 kDa)
p-ERK(Thr202/Tyr204)
(42, 44 kDa)
ERK (42, 44 kDa)
β-actin (43kDa)

Fig.3D

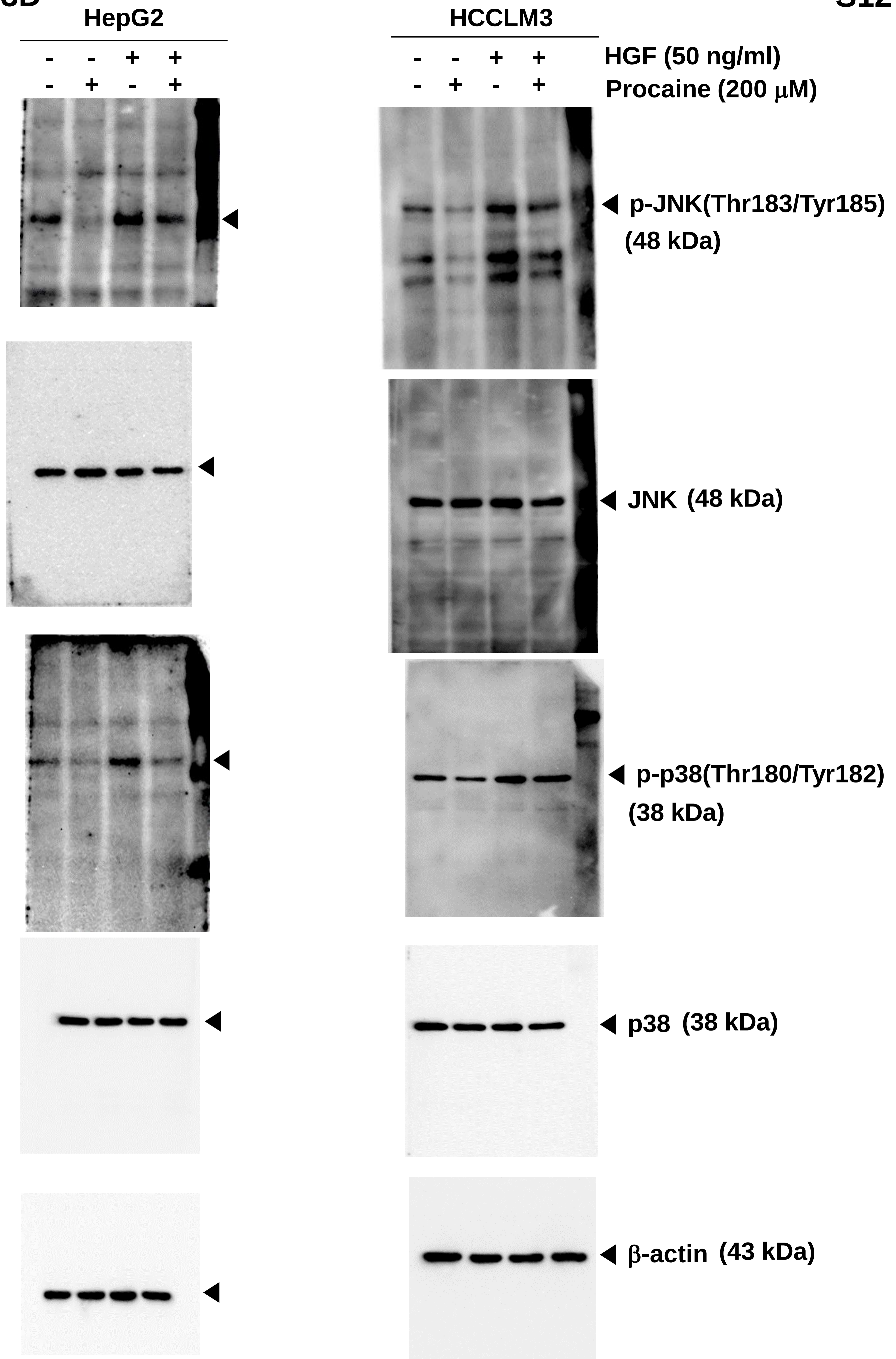

HepG2
HCCLM3
- - + +
- + - +
HGF (50 ng/ml)
Procaine (200 μM)
p-JNK(Thr183/Tyr185)
(48 kDa)
JNK (48 kDa)
p-p38(Thr180/Tyr182)
(38 kDa)
p38 (38 kDa)
β-actin (43 kDa)

Fig.5B

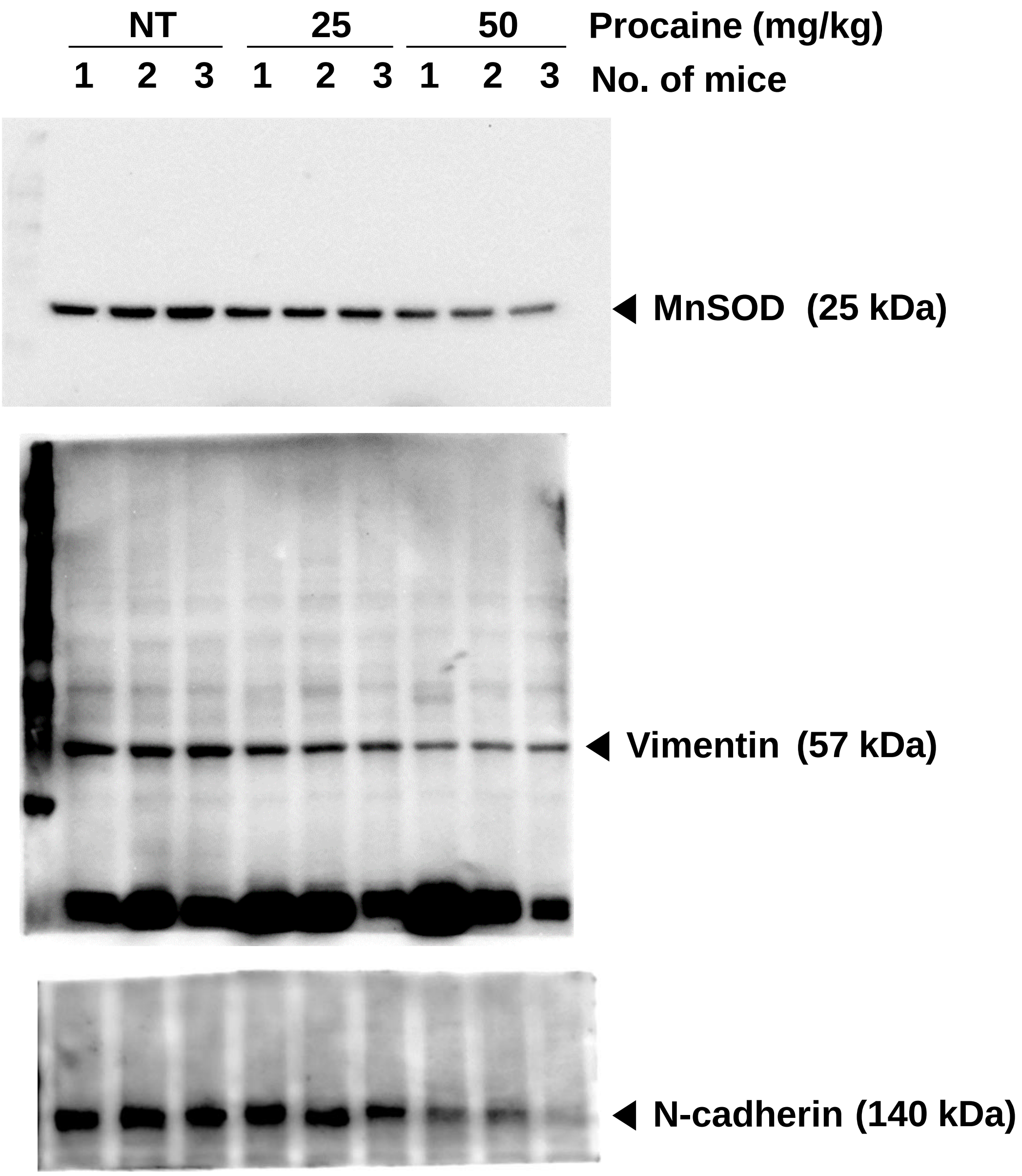

NT
25
50
Procaine (mg/kg)
1 2 3 1 2 3 1 2 3
No. of mice
MnSOD (25 kDa)
Vimentin (57 kDa)
N-cadherin (140 kDa)

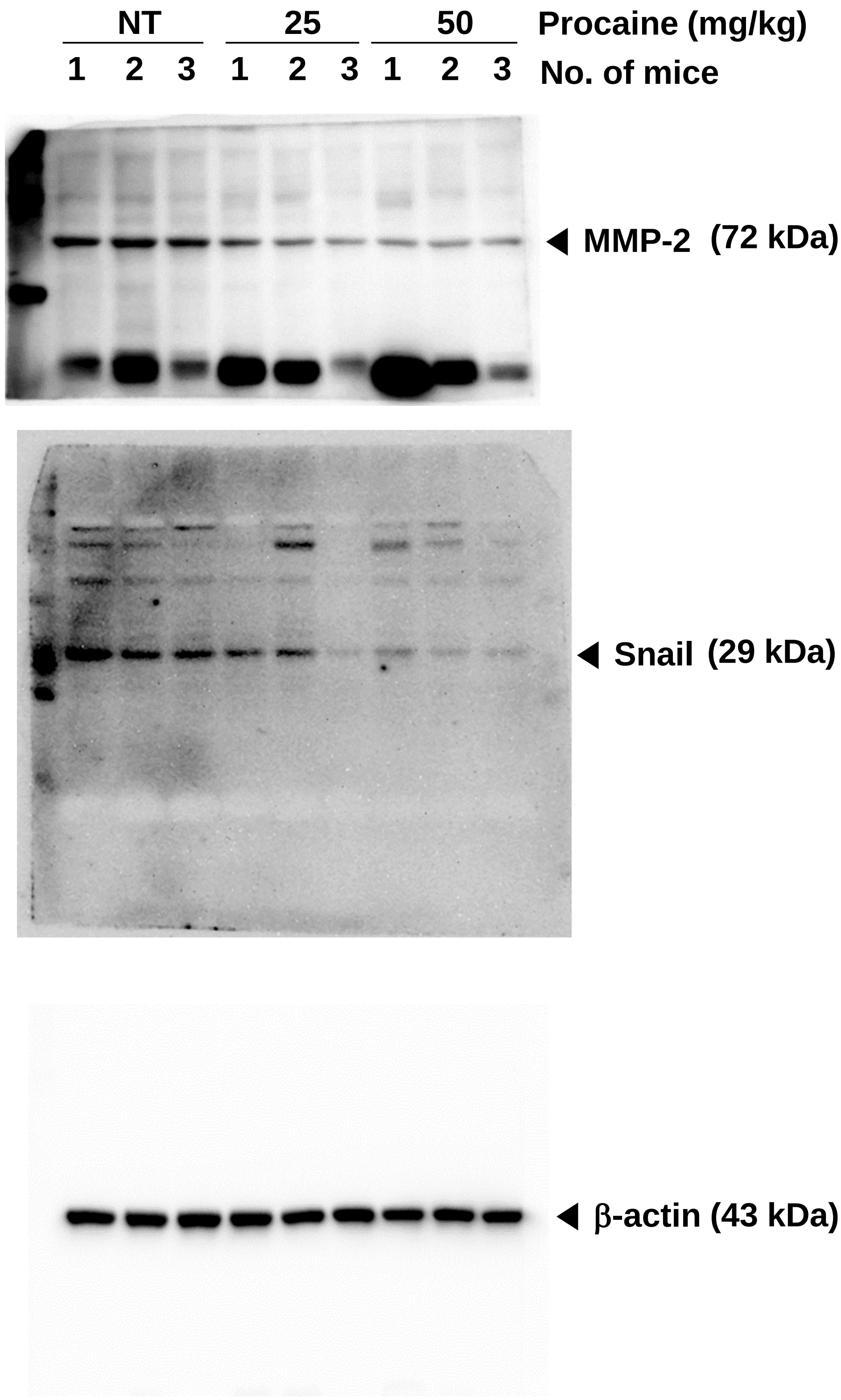
NT
25
50
Procaine (mg/kg)
1 2 3 1 2 3 1 2 3
No. of mice
MMP-2 (72 kDa)
Snail (29 kDa)
β-actin (43 kDa)

Fig.5B

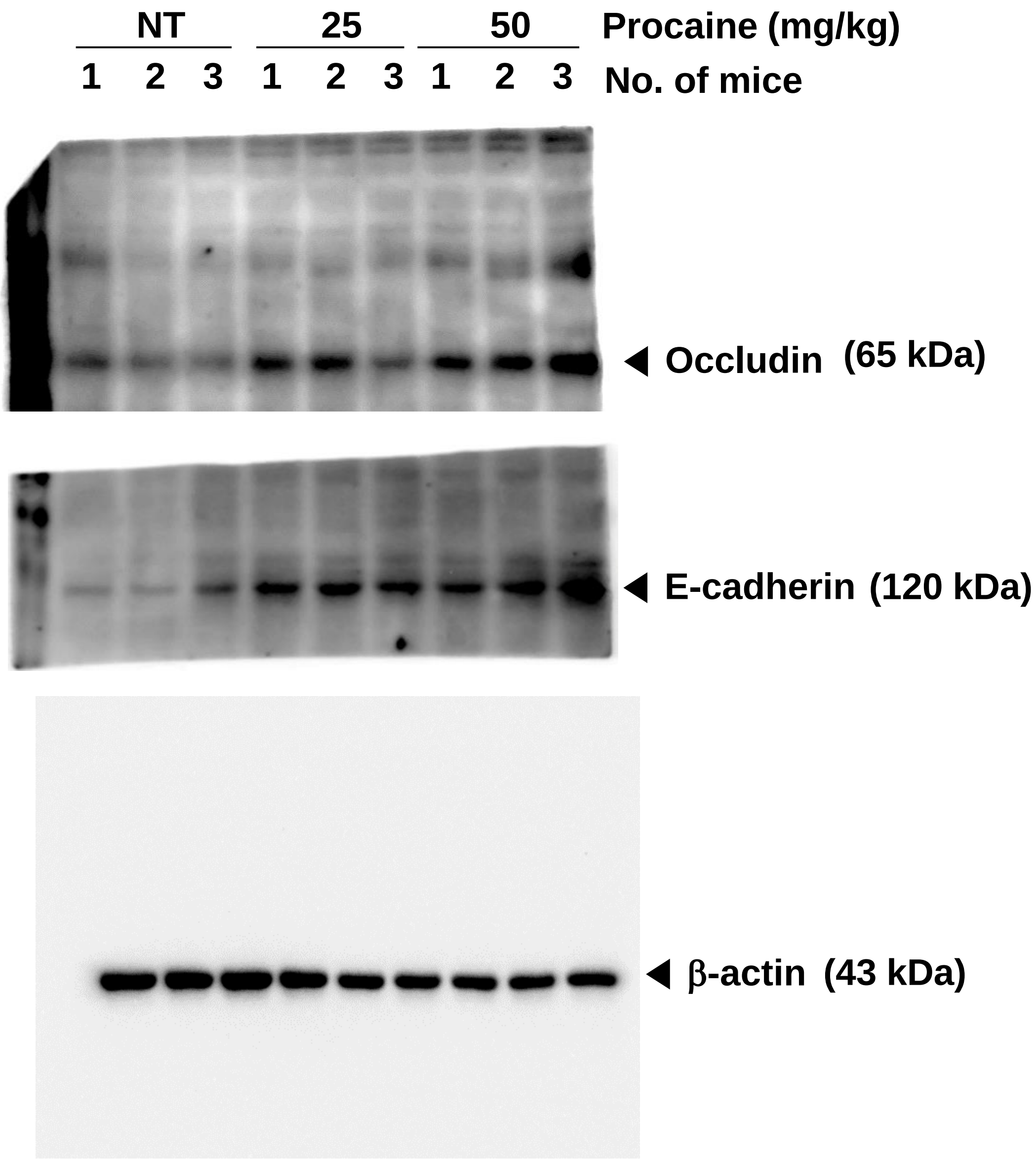

NT
25
50
Procaine (mg/kg)
1 2 3 1 2 3 1 2 3
No. of mice
Occludin (65 kDa)
E-cadherin (120 kDa)
β-actin (43 kDa)

Fig.5C

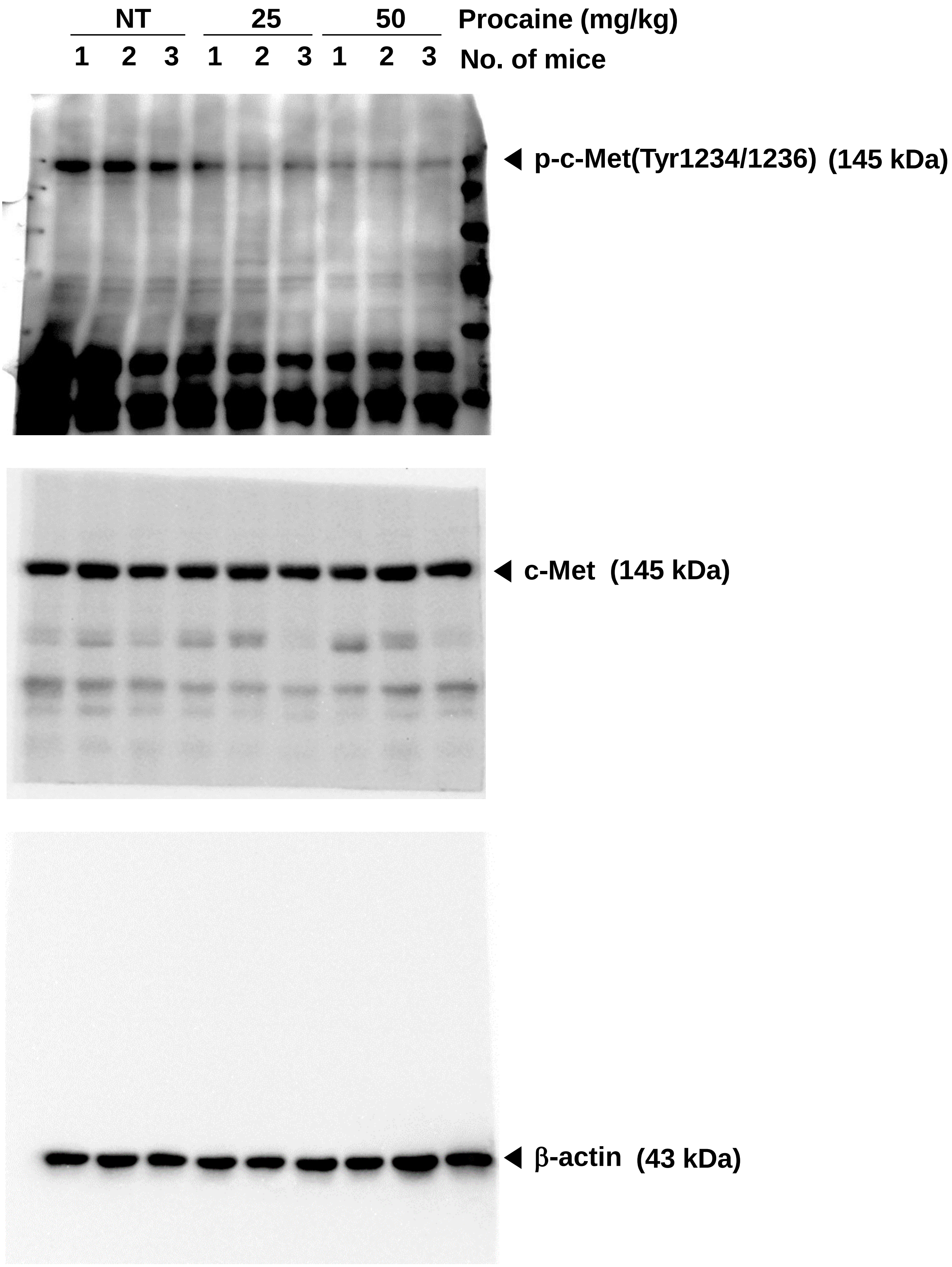
NT
25
50
Procaine (mg/kg)
1 2 3 1 2 3 1 2 3
No. of mice
p-c-Met(Tyr1234/1236) (145 kDa)
c-Met (145 kDa)
β-actin (43 kDa)

Fig.5D

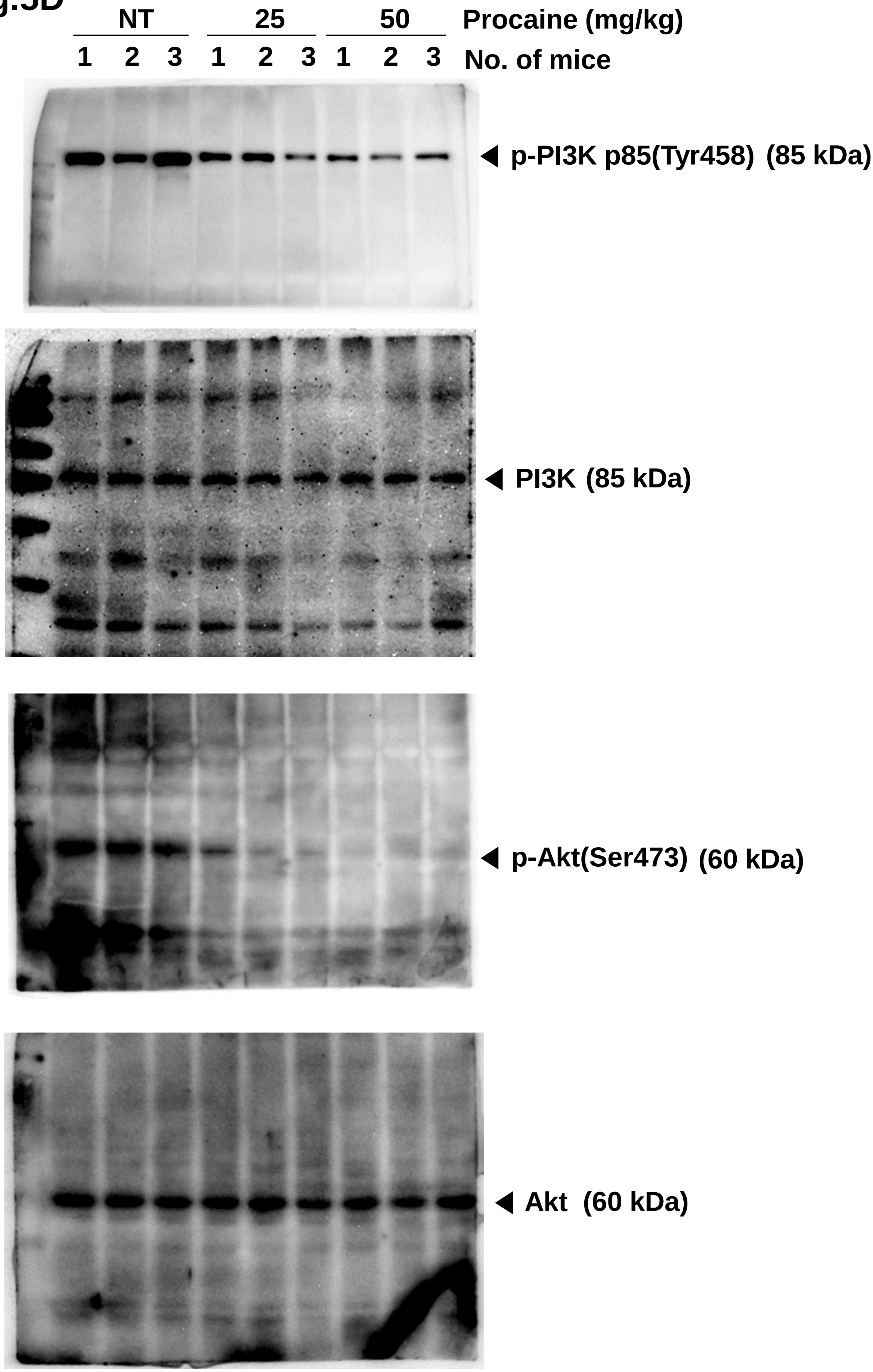
NT
25
50
Procaine (mg/kg)
1 2 3 1 2 3 1 2 3
No. of mice
p-PI3K p85(Tyr458) (85 kDa)
PI3K (85 kDa)
p-Akt(Ser473) (60 kDa)
Akt (60 kDa)

Fig.5D

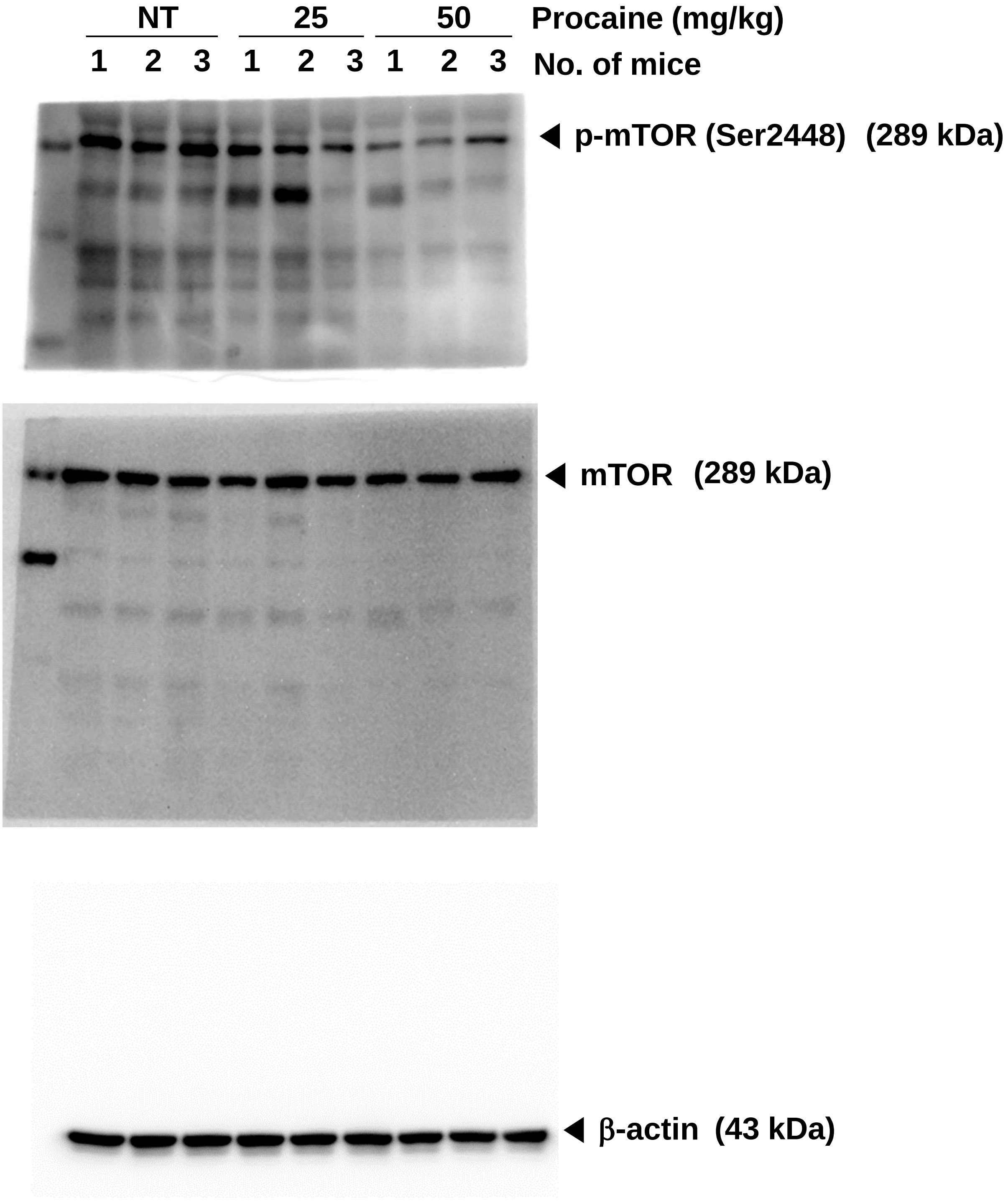
NT
25
50
Procaine (mg/kg)
1 2 3 1 2 3 1 2 3
No. of mice
p-mTOR (Ser2448) (289 kDa)
mTOR (289 kDa)
β-actin (43 kDa)

Fig.5D

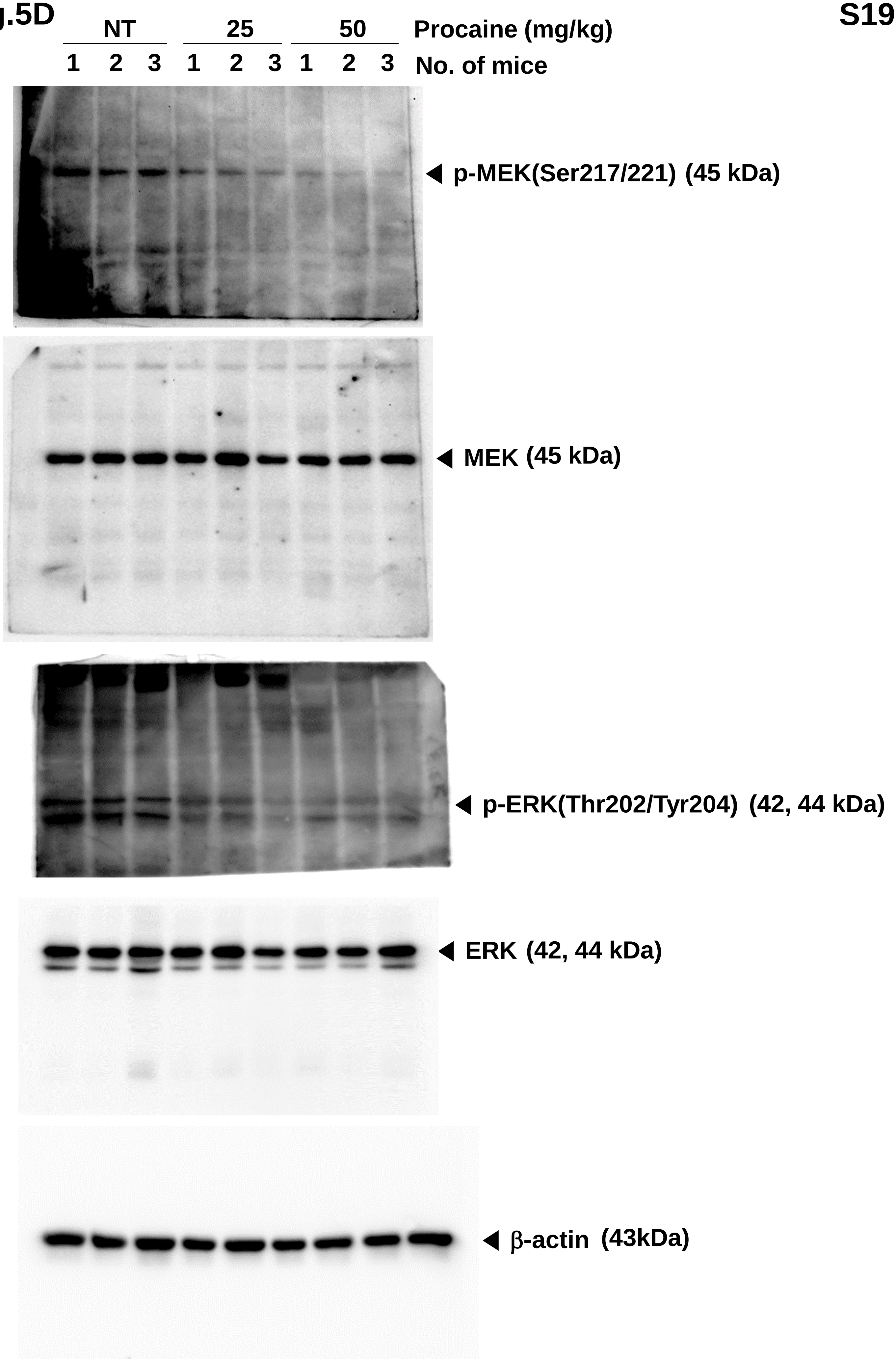
NT
25
50
Procaine (mg/kg)
1 2 3 1 2 3 1 2 3
No. of mice
p-MEK(Ser217/221) (45 kDa)
MEK (45 kDa)
p-ERK(Thr202/Tyr204) (42, 44 kDa)
ERK (42, 44 kDa)
β-actin (43kDa)
